# Does squatting need attention?—A dual-task study on cognitive resources in resistance exercise

**DOI:** 10.1371/journal.pone.0226431

**Published:** 2020-01-21

**Authors:** Fabian Herold, Dennis Hamacher, Alexander Törpel, Leonard Goldschmidt, Notger G. Müller, Lutz Schega

**Affiliations:** 1 Research Group Neuroprotection, German Center for Neurodegenerative Diseases (DZNE), Magdeburg, Germany; 2 Institute III, Department of Sport Science, Otto von Guericke University Magdeburg, Magdeburg, Germany; 3 Center for Behavioral Brain Sciences (CBBS), Magdeburg, Germany; 4 Department of Neurology, Medical Faculty, Otto von Guericke University Magdeburg, Magdeburg, Germany; Oregon Health and Science University, UNITED STATES

## Abstract

**Introduction:**

Accumulating evidence shows that acute resistance exercises and long-term resistance training positively influence cognitive functions, but the underlying mechanisms have been rarely investigated. One explanatory approach assumes that the execution of resistance exercises requires higher cognitive processes which, in turn, lead to an ‘indirect’ training of higher cognitive functions. However, current knowledge on the engagement of higher cognitive functions during the execution of resistance exercises is relatively sparse. Hence, the purpose of this study was to examine to what extent cognitive resources are needed to perform a resistance exercise in the form of barbell back squatting.

**Methods:**

Twenty-four young adults performed a cognitive task (serial subtraction of 7’s) during standing and during barbell back squatting on a Smith machine. The total number and the number of correct responses were analyzed and taken as indicators of the cognitive load imposed by the experimental condition (squatting) and the control condition (standing). Additionally, participants’ perceived exertion, mean heart rate, and the number of squats they were able to perform were assessed.

**Results:**

While accuracy scores were found not to be significantly different between conditions, the numbers of total and of correct responses were significantly lower during squatting than during standing. Additionally, during squatting a higher number of total answers was given in the fifth set compared to the first set. We attribute this phenomenon to a learning effect. Furthermore, there was no statistically significant correlation between cognitive measures and perceived exertion.

**Conclusion:**

Results suggest that perceived exertion cannot explain the higher dual-task costs observed during squatting. They rather reflect that more cognitive resources are needed to perform low-load barbell back squats than during standing. However, further research is necessary to confirm and generalize these findings.

## Introduction

There is growing evidence in the literature that acute resistance exercises and long-term resistance training improve cognitive functions [1–4]. However, the underlying mechanisms for these cognitive improvements are not fully understood yet, although they seem to rely on changes at multiple levels [5–7]. One assumption is that resistance exercises may act as an ‘indirect’ form of cognitive training since for their execution subjects need to constantly engage cognitive resources as they have to pay attention to perform the movement with an appropriate technique (e.g., squat), to produce an appropriate level of force, and to observe the surroundings in order not to harm themselves or others [3]. Engaging specific cognitive resources to execute a specific motor task (e.g., resistance exercise such as squatting) is deemed a necessary prerequisite to guide the facilitation effects of physical exercises. The latter provides the basis for cognitive improvements in response to physical training interventions [8]. However, to our current knowledge, there is currently no study that investigated the cognitive resources needed to execute dynamic resistance exercises (e.g., squats). Therefore, the assumption that resistance exercises ‘indirectly’ train cognitive functions due to the engagement of cognitive resources requires further exploration.

An established behavioral approach to quantify the cognitive resources which are needed to execute a motor task is the dual-task paradigm. For instance, the dual-task paradigm is frequently utilized to investigate the amount of cognitive resources required during walking or postural tasks [9–13]. Using the dual-task paradigm, an individual’s performance during a single-task condition (e.g., performing a cognitive task) is compared with his/her performance during a dual-task condition (e.g., performing a motor task [e.g., squatting] and a cognitive task simultaneously). The changes in performance from single-task to dual-task (also known as dual-task costs) are used to probe the amount of cognitive resources needed to execute the motor task (e.g., squatting). In this study, we aimed to investigate whether higher cognitive resources are required to perform barbell back squatting. To do so, a dual-task paradigm was applied and the relative increase in cognitive resources needed to perform the resistance exercise ‘barbell back squats’ was examined.

## Materials and methods

Twenty-four (10f /14m) healthy adults participated in this randomized study (mean age (± SD): 24.38 ± 3.15 years; mean height: 173.92 ± 8.29 cm; mean body mass: 70.25 ± 11.56 kg). All study procedures were in accordance with the Declaration of Helsinki (1964) and were approved by the local ethics committee of the Medical Faculty of the Otto von Guericke University Magdeburg (181/18). Each participant was asked to visit the laboratories for two sessions at least 48 hours apart. At the first session, the participants were informed about the experimental procedures and had to complete the German version of the Physical Activity Readiness Questionnaire (PARQ) which screens for individuals at increased health risk when exercising physically [14–16]. All individuals interested in participating in this study verified by self-reports that they were not suffering from musculoskeletal, cardiovascular, and/or neurological disorders. Based on the results of the PARQ and self-reports, individuals at an increased health risk while exercising were excluded from this study. Furthermore, all participants gave their written informed consent to participate in this study and received a compensation of 24€.

In addition, all participants completed the Minimal Mental State Examination (MMSE) [17], Trail Making Test (TMT A&B) [18], the Beck Depression Inventory (BDI-II) [19], and a physical activity questionnaire (BSA; derived from the German Bewegungs- und Sportaktivitätsfragebogen) [20] at their first visit. The MMSE consists of 11 items and screens for cognitive impairments (indicated by lower scores) [17]. The TMT A is considered to measure abilities of visual search while TMT B quantifies the performance of higher cognitive abilities such as cognitive flexibility [21,22]. The difference between the performance of TMT B and TMT A is presumed to be a measure of shifting ability [23]. BDI-II reflects a measure of depressive symptoms whereas higher scores reflect a higher severity of depression [19]. The BSA measures the level of physical activity and physical exercise engaged within the last four weeks [20]. Additionally, the participants’ experience in resistance training was quantified using a visual analogue scale ranging from 0 (i.e., no experience) to 100 (i.e., strong experience). After the completion of the questionnaires, a standardized warm-up was conducted to prepare the participants for a one-repetition maximum (1-RM) test. The warm-up consisted of five minutes of stationary cycling (1 W per kilogram body weight at 60 to 80 revolution per minute) and one set of barbell back squats with ten repetitions at light loads [24]. Then, based on established testing protocols [25], several sets of barbell back squats were performed until a load was found which the participants could lift exactly one time with a proper technique (stance width: shoulder width / squat depth: at least horizontal thighs). Between the testing sets, participants rested for at least three minutes and immediately after each set the perceived exertion was quantified using the Repetitions in Reserve scale (RIR) [26]. The RIR was used as countermeasure to verify that the 1-RM is the maximal load that the participants can lift. The 1-RM of the participants was determined within four (± 1) sets and the corresponding mean RIR was ten. The barbell back squats were performed using a Smith machine (integrated in the squat rack by MAXXUS^®^; version 9.1).

**Fig 1:**
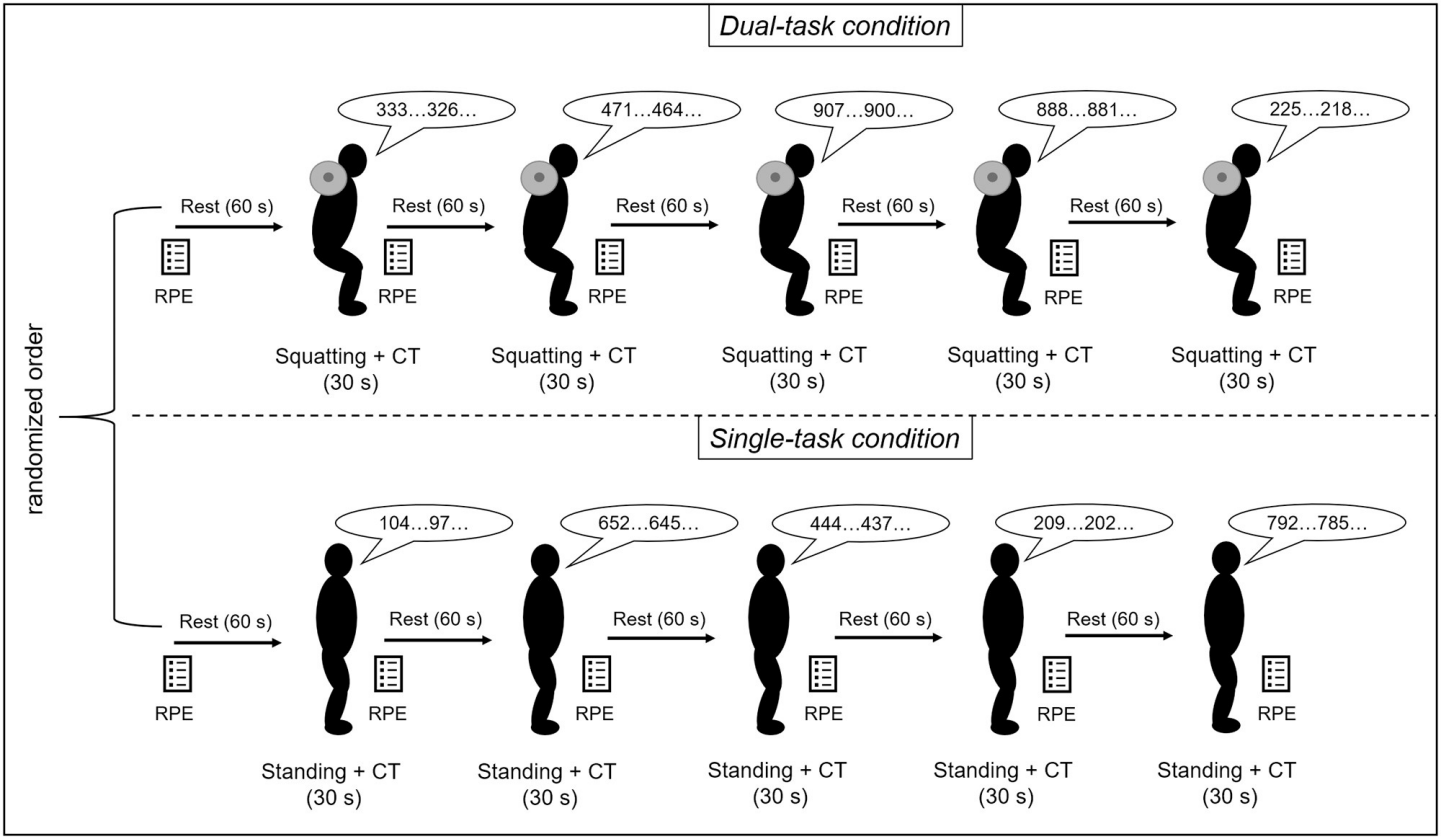
Overview of the experimental protocol in the second session and the time points of the assessment of RPE. The cognitive task was solved in a standing position (single-task) and during squatting (dual-task). Both tasks were performed for 30 seconds and afterwards the participants rested in a standing position for 60 seconds. RPE: Rating of perceived exertion.

During the second visit, the participants performed a single-task condition (solving a cognitive task during standing, [ST]) and a dual-task condition (squatting while solving a cognitive task, [DT]). The conditions were separated by a rest period of ten minutes and conducted in a randomized order (balanced permuted block randomization) by using the Web site ‘Randomization.com’ (http://www.randomization.com). To make ST and DT comparable, the time of each set was limited to 30 seconds and between the sets, a rest in a standing position of 60 seconds was given (see Fig 1). In this study, a set is defined as the 30 seconds in which the participants solve the cognitive tasks while standing or performing barbell back squats. In DT ten seconds just before the end of the rest period, a cue was given that enable the participants to take the starting position of the barbell back squats to make sure that they had have the full 30 seconds to solve the cognitive task as in ST. After finishing the set in DT, the barbell was placed back into the squat rack. In DT the participants were allowed to squat with their preferred repetition velocity. In each condition, five sets were conducted and in DT the load of the barbell was set to of 40% of 1-RM. We choose 40% of 1-RM because it was shown that even this low load leads to cognitive improvements [27]. As cognitive task, we used the serial subtraction of 7’s from a three-digit number [28]. A new, randomly assigned three-digit number was given to the participants at the beginning of each set in ST and DT. Furthermore, as shown in Fig 1, the rating of perceived exertion (RPE) using a RPE scale which ranged from 6 (no exertion) to 20 (maximal exertion) was administered during the second visit [29].

Furthermore, we measured mean heart rate (HR) continuously with a portable heart rate (HR) monitor (V800, Polar Electro Oy^®^, Kempele, Finland) and analyzed HR data using ‘Kubios HRV’ (Biosignal Analysis and Medical Imaging Group, Universität Kuopio, Finland; Version 3.3.1) [30]. In Kubios artefacts were removed by applying the threshold-based artefact correction algorithm. Therefore, we set the threshold to a medium level (i.e., values that differ more than 0.25 s from average were replaced with interpolated values using a cubic spline interpolation) [30–32]. Furthermore, the HR time series was detrended by using the smoothness-priors-based detrending approach (smoothing parameter, λ = 500) [30]. Thereafter, the mean heart rate was calculated from corrected and detrended HR time series using whole 30 seconds of task periods (i.e., Squatting + DT and Standing + DT; see Fig 1) and the middle 30 seconds of the first rest period (for ‘Pre’; see Fig 1 and Table 1).

**Table 1:** Personal data for the characterization of the participants and results of the screening test in the investigated sample; BDI: Becks Depression Inventory; BSA: Physical activity questionnaire, derived from German ‘Bewegungs- und Sportaktivitätsfragebogen’; MMSE: Minimal Mental State Examination; PA: Physical activity; PE: Physical exercise; 1-RM: One-repetition maximum; TMT: Trail Making Test.

Parameters	Mean ± SD
Years of education [years]	15.8 ± 3.0
MMSE score	29.60 ± 0.63
BDI-II	3.29 ± 3.43
BSA [min per week]	PA: 313.60 ± 237.33 / PE: 338.95 ± 223.25
TMT A time [sec] / errors	20.87 ± 5.16 / 0.04 ± 0.20
TMT B time [sec] / errors	41.46 ± 9.25 / 0.08 ± 0.40
TMT B-A time [sec]	20.59 ± 7.77
1-RM [kg]	97.70 ± 27.26
1-RM normalized to body mass	1.39 ± 0.41
Self-rated experience in resistance training	49.79 ± 25.43
Resistance training sessions per week	2.06 ± 1.69

Additionally, during the sets in DT the number of squat repetitions was counted.

### Statistical analysis

The statistical analysis was performed using IBM SPSS (Statistical Package for social science, Version 22, Chicago, IL, USA) and non-parametric tests were conducted because not all data were normally distributed. To compare performance in single-task conditions versus performance in dual-task conditions, the Wilcoxon test was performed. To identify a possible main effect of time (respectively set), a Friedman test with post-hoc analyses (i.e., Wilcoxon tests) were conducted. Outliers were not removed from statistical analyses because non-parametric test are relatively robust against the effects of those [33,34]. The effect sizes for the Wilcoxon tests were calculated using following formula $r=\frac{\left|z\right|}{\sqrt{N}}$ and were rated as follows: 0.5 large effect, 0.3 medium effect, and 0.1 small effect [35,36].

Furthermore, in order to examine possible relationships between cognitive measures and RPE, mean HR, number of squat repetitions, or self-rated experience in resistance training, correlation analyses were performed. Therefore, Spearman’s Rho (r_s_) was calculated and rated as follows: 0.00 to 0.19 no correlation; 0.20 to 0.39 low correlation; 0.40 to 0.59 moderate correlation; 0.60 to 0.79 moderately high correlation; ≥ 0.8 high correlation [37]. The level of significance was initially set to α = 0.05 for all statistical analyses. In order to account for the multiple comparison problem in post-hoc tests and correlation analyses (correction within one tested condition between the five sets), the Holm correction method was applied [38]. Therefore, the *n* p_raw_-values (where *n* is the number of p_raw_-values corresponding to one hypothesis) were ordered in an ascending order starting with the smallest p_raw_-value (p_raw(1)_, …, p_raw(n)_). Afterwards, the ordered p_raw_-values are compared to the threshold α_j_ calculated as follows: p_(i)_ ≤ α_j_ = α/(*n* − (*i* -1)). The Holm correction will stop at the i^th^ test for which the first non-rejection occurs (i.e., the *i* for which p_raw(i)_ > α_i_) [38,39]. Furthermore, the corrected p-values (p_corrected_) were calculated by using the following formula [40,41]: p_corrected_ = p_(i)raw_ × /(*n* − (*i* -1)). Please note that for the calculation of p_corrected_ the p_raw values_ were ordered as described in Holm correction.

## Results

The general characteristics of the participants are displayed in Table 1.

### Cognitive measures

A descriptive overview about the number of total answers and number of correct answers in ST and DT for the task serial subtraction of 7’s is provided in Table 2.

**Table 2:** Median (interquartile range) of cognitive measures and RPE in single-task condition and dual-task condition are shown. Additionally, the number of repetitions of barbell back squats in dual-task condition is presented.

Parameter	Pre	1^st^ set	2^nd^ set	3^rd^ set	4^th^ set	5^th^ set
**Single-task condition**						
Total number of answers	n.a.	12.0 (5.0) *	12.0 (6.0) *	14.0 (5.0) *	12.0 (5.0) *	13.0 (5.0) *
Number of correct answers	n.a.	12.0 (7.0) *	12.0 (8.0) *	13.0 (6.0) *	12.0 (5.0) *	12.5 (5.0) *
Accuracy score (in %)	n.a.	100.0 (9.1)	100.0 (0.0)	100.0 (9.2)	100.0 (0.0)	100.0 (7.7)
RPE score	6.0 (1.0) ^b^	7.0 (3.0) *^,^ ^b^	8.0 (4.0) *^,^ ^b^	7.5 (3.0) *^,^ ^b^	7.5 (4.0) *^,^ ^b^	8.0 (4.0) *^,^ ^b^
Mean HR	87.0 (19.5)	92.5 (24.3) *^,^ ^b^	86.0 (22.8) *	87.0 (23.3) *	86.5 (20.5) *	87.5 (25.0) *
**Dual-task condition**						
Total number of answers	n.a.	9.0 (4.0) *^,^ ^a^	10.0 (3.0) *	10.0 (5.0) *	11.0 (4.0) *	11.0 (4.0) *^,^ ^a^
Number of correct answers	n.a.	9.0 (3.0) *	9.5 (4.0) *	9.5 (4.0) *	10.5 (5.0) *	10.0 (4.0) *
Accuracy score (in %)	n.a.	100.0 (9.1)	100.0 (10.0)	96.2 (11.1)	100.0 (12.2)	100.0 (9.8)
RPE score	6.0 (1.0) ^b^	13.0 (2.0) *^,^ ^b^	13.0 (1.0) *^,^ ^b^^,^ ^c^	14.0 (2.0) *^,^ ^b^^,^ ^c^^,^ ^d^	15.0 (3.0) *^,^ ^b^^,^ ^c^^,^ ^d^^,^ ^e^	15.5 (3.0) *^,^ ^b^^,^ ^c^^,^ ^d^^,^ ^e^^,^ ^f^
Mean HR	80.5 (17.5)	121.0 (17.3) *^,^ ^b^	127.5 (25.8) *^,^ ^b^^,^ ^c^	130.5 (34.0) *^,^ ^b^^,^ ^c^^,^ ^d^	131.0 (36.3) *^,^ ^b^^,^ ^c^^,^ ^d^^,^ ^e^	139.5 (41.0) *^,^ ^b^^,^ ^c^^,^ ^d^^,^ ^e^^,^ ^f^
Number of squat repetitions	n.a.	11.0 (3.0)	11.0 (2.0)	12.0 (3.0)	12.0 (2.0)	12.0 (2.0)

^a^: indicates a significant difference between 1^st^ and 5^th^ set;

^b^: indicates significant difference between ‘Pre’ and sets;

^c^: indicates significant difference between 1^st^ set and the respective set;

^d^: indicates significant difference between 2^nd^ set and the respective set;

^e^: indicates significant difference between 3^rd^ set and the respective set;

^f^: indicates significant difference between 4^th^ set and respective set;

n.a.: not applicable; HR: heart rate; RPE: Rating of relative perceived exertion.

*: indicates a significant difference between single-task condition and dual-task-condition.

To evaluate the effect of DT on the number of total answers and number of correct answers, performance in DT was compared to the performance in ST.

With regard to the number of total answers in the first set (Z (N = 24) = -3.759, p_corrected_ = 0.001; r = 0.77), the second set (Z (N = 24) = -3.462, p_corrected_ = 0.002; r = 0.71), the third set (Z (N = 24) = -3.505, p_corrected_ = 0.002; r = 0.72), the fourth set (Z (N = 24) = -3.034, p_corrected_ = 0.005; r = 0.62), and the fifth set (Z (N = 24) = -2.414, p_corrected_ = 0.016; r = 0.50) a lower number of total answers was given in DT compared to ST.

The statistical comparison concerning the number of correct answers shows that in the first set (Z (N = 24) = -3.324, p_corrected_ = 0.003; r = 0.68), the second set (Z (N = 24) = -3.576, p_corrected_ = 0.001; r = 0.73), the third set (Z (N = 24) = -3.633, p_corrected_ = 0.001; r = 0.74), the fourth set (Z (N = 24) = -3.304, p_corrected_ = 0.002; r = 0.67), and the fifth set (Z (N = 24) = -2.476, p_corrected_ = 0.013; r = 0.51) a lower number of correct answers was given in DT compared to ST.

Furthermore, a significant effect of time was observed for the number of total answers in DT (X^2^ = 13.741 (df = 4, n = 24), p = 0.008) but not in ST. After post-hoc tests and the following Holm adjustment, it was observed that in DT in the fifth set a higher number of total answers was given compared to the first set (Z (N = 24) = -3.143, p_corrected_ = 0.017; r = 0.64).

A significant effect of time was registered for correct answers in DT (X^2^ = 10.047 (df = 4, n = 24), p = 0.040) but not in ST. However, the effects observed in post-hoc tests in DT did not remain their statistical significance after the application of Holm correction method.

With regard to the accuracy score, we did neither observe significant differences between ST and DT (p_corr_ > 0.05) nor was a statistically significant effect of time in ST (X^2^ = 6.194 (df = 4, n = 24), p = 0.185) and in DT (X^2^ = 0.712 (df = 4, n = 24), p = 0.950) noticed.

### Psychophysiological measures

#### Ratings of perceived exertion

A descriptive overview about RPE ratings obtained in ST and DT is provided in Table 2. The difference between RPE values obtained prior to ST or DT were not statistically significant (Z (N = 24) = 0.000, p_corrected_ = 1.000; r = 0.00), whereas after first set (Z (N = 24) = -4.214, p_corrected_ < 0.001; r = 0.86), second set (Z (N = 24) = -4.296, p_corrected_ < 0.001; r = 0.88), third set (Z (N = 24) = -4.295, p_corrected_ < 0.001; r = 0.88), fourth set (Z (N = 24) = -4.295, p_corrected_ < 0.001; r = 0.88), and fifth set (Z (N = 24) = -4.291, p_corrected_ < 0.001; r = 0.88) the RPE score were significantly larger in DT (see Table 2).

In the ST condition, a main effect of time was observed (X^2^ = 36.684 (df = 5, n = 24), p > 0.001) and post-hoc test indicate that the RPE scores obtained prior the sets was significantly lower than after first set (Z (N = 24) = -2.953, p_corrected_ = 0.035; r = 0.60), second set (Z (N = 24) = -3.324, p_corrected_ = 0.012; r = 0.68), third set (Z (N = 24) = -3.219, p_corrected_ = 0.015; r = 0.66), fourth set (Z (N = 24) = -3.453, p_corrected_ = 0.008; r = 0.70), and fifth set (Z (N = 24) = -3.321, p_corrected_ = 0.012; r = 0.68). Between the sets no significant changes in RPE scores were observed (p_corrected_ > 0.05).

In the DT condition we observed a significant main effect of time (X^2^ = 104.526 (df = 5, n = 24), p < 0.001). The post-hoc analyses show that the RPE score obtained prior the sets was lower than after first set (Z (N = 24) = -4.219, p_corrected_ < 0.001; r = 0.86), second set (Z (N = 24) = -4.313, p_corrected_ < 0.001; r = 0.88), third set (Z (N = 24) = -4.308, p_corrected_ < 0.001; r = 0.88), fourth set (Z (N = 24) = -4.299, p_corrected_ < 0.001; r = 0.88) and fifth set (Z (N = 24) = -4.295, p_corrected_ < 0.001; r = 0.88). Furthermore, following statistically significant changes between sets were observed: first set vs. second set (Z (N = 24) = -3.466, p_corrected_ = 0.002; r = 0.71), first set vs. third set (Z (N = 24) = -3.685, p_corrected_ = 0.001; r = 0.75), first set vs. fourth set (Z (N = 24) = -4.140, p_corrected_ < 0.001; r = 0.85), first set vs. fifth set (Z (N = 24) = -4.220, p_corrected_ < 0.001; r = 0.86), second set vs. third set (Z (N = 24) = -2.758, p_corrected_ = 0.006; r = 0.56), second set vs. fourth set (Z (N = 24) = -4.008, p_corr_ < 0.001; r = 0.82), second set vs. fifth set (Z (N = 24) = -4.162, p_corrected_ < 0.001; r = 0.85), third set vs. fourth set (Z (N = 24) = -3.499, p_corrected_ = 0.002; r = 0.71), third set vs. fifth set (Z (N = 24) = -3.858, p_corrected_ = 0.001; r = 0.79), and forth set vs. fifth set (Z (N = 24) = -3.211, p_corrected_ = 0.003; r = 0.66).

#### Mean heart rate

The difference between mean HR obtained prior to ST or DT were not statistically significant (Z (N = 24) = -1.121, p_corrected_ = 0.262; r = 0.23), whereas during the first set (Z (N = 24) = -4.258, p_corrected_ < 0.001; r = 0.87), second set (Z (N = 24) = -4.286, p_corrected_ < 0.001; r = 0.87), third set (Z (N = 24) = -4.288, p_corrected_ < 0.001; r = 0.88), fourth set (Z (N = 24) = -4.287, p_corrected_ < 0.001; r = 0.88), and fifth set (Z (N = 24) = -4.287, p_corrected_ < 0.001; r = 0.88) the mean HR was significantly larger in DT (see Table 2).

Furthermore, we observed in ST a significant main effect of time (X^2^ = 15.524 (df = 5, n = 24), p = 0.008). The post-hoc analyses show that the mean HR obtained prior to the sets was lower than during the first set (Z (N = 24) = -2.992, p_corrected_ = 0.042; r = 0.61).

In DT we observed a significant main effect of time regarding the change in mean HR (X^2^ = 96.037 (df = 5, n = 24), p < 0.001). The post-hoc analyses showed that the mean HR score obtained prior the sets was lower than during the first set (Z (N = 24) = -4.292, p_corrected_ < 0.001; r = 0.88), second set (Z (N = 24) = -4.286, p_corrected_ < 0.001; r = 0.87), third set (Z (N = 24) = -4.287, p_corrected_ < 0.001; r = 0.88), fourth set (Z (N = 24) = -4.286, p_corrected_ < 0.001; r = 0.87) and fifth set (Z (N = 24) = -4.287, p_corrected_ < 0.001; r = 0.88). The following statistically significant changes were observed: first set vs. second set (Z (N = 24) = -3.409, p_corrected_ = 0.003; r = 0.70), first set vs. third set (Z (N = 24) = -3.653, p_corrected_ = 0.001; r = 0.75), first set vs. fourth set (Z (N = 24) = -3.803, p_corrected_ = 0.001; r = 0.78), first set vs. fifth set (Z (N = 24) = -4.201, p_corrected_ < 0.001; r = 0.86), second set vs. third set (Z (N = 24) = -3.036, p_corrected_ = 0.005; r = 0.62), second set vs. fourth set (Z (N = 24) = -3.361, p_corrected_ = 0.002; r = 0.69), second set vs. fifth set (Z (N = 24) = -4.124, p_corrected_ < 0.001; r = 0.84), third set vs. fourth set (Z (N = 24) = -2.910, p_corrected_ = 0.004; r = 0.60), third set vs. fifth set (Z (N = 24) = -4.033, p_corrected_ < 0.001; r = 0.82), fourth set vs. fifth set (Z (N = 24) = -3.983, p_corrected_ < 0.001; r = 0.81).

### Number of repetitions

Regarding the number of repetitions, there was no significant differences between the sets (main time effect: X^2^ = 8.846 (df = 4, n = 24), p = 0.065) and, hence, no post-hoc tests were performed.

### Correlation between specific outcome variables

We neither found statistically significant correlations between measures of cognition (i.e., total number of answers, number of correct answers, and accuracy score) and RPE or mean HR in each of the five sets nor were there statistically significant correlations between measures of cognition and self-rated experience in resistance training or resistance training sessions per week (see Table 3). A significant moderate positive correlation between number of squats and total number of correct answers was observed in the first set of DT condition (see Table 3).

**Table 3:** Overview of correlation coefficients (Spearman’s Rho [r_s_]) and corresponding p-values / RPE: Rating of relative perceived exertion; sERT: Self-rated experience in strength training; Reps: Number of squat repetitions; RTS: Resistance training sessions per week.

Correlation	1^st^ set	2^nd^ set	3^rd^ set	4^th^ set	5^th^ set
**Single-task condition**					
RPE and total number of answers	r_s_ = -0.19 (p_corrected_ = 1.00)	r_s_ = -0.13 (p_corrected_ = 1.00)	r_s_ = -0.04 (p_corrected_ = 0.84)	r_s_ = -0.08 (p_corrected_ = 1.00)	r_s_ = -0.17 (p_corrected_ = 1.00)
RPE and number of correct answers	r_s_ = -0.14 (p_corrected_ = 1.00)	r_s_ = -0.11 (p_corrected_ = 1.00)	r_s_ = -0.04 (p_corrected_ = 0.86)	r_s_ = -0.08 (p_corrected_ = 1.00)	r_s_ = -0.12 (p_corrected_ = 1.00)
RPE and accuracy score	r_s_ = -0.30 (p_corrected_ = 1.00)	r_s_ = -0.08 (p_corrected_ = 1.00)	r_s_ = -0.01 (p_corrected_ = 1.00)	r_s_ = 0.05 (p_corrected_ = 1.00)	r_s_ = 0.14 (p_corrected_ = 1.00)
Mean HR and total number of answers	r_s_ = -0.00 (p_corrected_ = 0.99)	r_s_ = 0.06 (p_corrected_ = 1.00)	r_s_ = 0.06 (p_corrected_ = 1.00)	r_s_ = 0.13 (p_corrected_ = 1.00)	r_s_ = 0.17 (p_corrected_ = 1.00)
Mean HR and number of correct answers	r_s_ = -0.03 (p_corrected_ = 1.00)	r_s_ = 0.06 (p_corrected_ = 1.00)	r_s_ = 0.01 (p_corrected_ = 0.96)	r_s_ = 0.08 (p_corrected_ = 1.00)	r_s_ = 0.09 (p_corrected_ = 1.00)
Mean HR and accuracy score	r_s_ = -0.03 (p_corrected_ = 1.00)	r_s_ = -0.02 (p_corrected_ = 0.94)	r_s_ = -0.31 (p_corrected_ = 0.57)	r_s_ = -0.28 (p_corrected_ = 0.56)	r_s_ = -0.44 (p_corrected_ = 0.17)
**Dual-task condition**					
RPE and total number of answers	r_s_ = -0.23 (p_corrected_ = 0.84)	r_s_ = -0.43 (p_corrected_ = 0.15)	r_s_ = -0.21 (p_corrected_ = 0.33)	r_s_ = -0.35 (p_corrected_ = 0.40)	r_s_ = -0.21 (p_corrected_ = 0.64)
RPE and number of correct answers	r_s_ = -0.22 (p_corrected_ = 1.00)	r_s_ = -0.40 (p_corrected_ = 0.25)	r_s_ = -0.11 (p_corrected_ = 1.00)	r_s_ = -0.22 (p_corrected_ = 0.93)	r_s_ = -0.07 (p_corrected_ = 0.76)
RPE and accuracy score	r_s_ = -0.00 (p_corrected_ = 1.00)	r_s_ = 0.30 (p_corrected_ = 1.00)	r_s_ = 0.13 (p_corrected_ = 1.00)	r_s_ = 0.34 (p_corrected_ = 0.53)	r_s_ = -0.01 (p_corrected_ = 1.00)
Mean HR and total number of answers	r_s_ = 0.37 (p_corrected_ = 0.40)	r_s_ = 0.25 (p_corrected_ = 0.25)	r_s_ = 0.32 (p_corrected_ = 0.40)	r_s_ = 0.31 (p_corrected_ = 0.28)	r_s_ = 0.37 (p_corrected_ = 0.32)
Mean HR and number of correct answers	r_s_ = 0.36 (p_corrected_ = 0.44)	r_s_ = 0.16 (p_corrected_ = 0.45)	r_s_ = 0.28 (p_corrected_ = 0.76)	r_s_ = 0.22 (p_corrected_ = 0.59)	r_s_ = 0.23 (p_corrected_ = 0.86)
Mean HR and accuracy score	r_s_ = -0.17 (p_corrected_ = 1.00)	r_s_ = -0.23 (p_corrected_ = 1.00)	r_s_ = 0.11 (p_corrected_ = 1.00)	r_s_ = -0.02 (p_corrected_ = 0.92)	r_s_ = -0.08 (p_corrected_ = 1.00)
sERT and total number of answers	r_s_ = 0.30 (p_corrected_ = 0.80)	r_s_ = 0.14 (p_corrected_ = 1.00)	r_s_ = -0.08 (p_corrected_ = 1.00)	r_s_ = 0.01 (p_corrected_ = 1.00)	r_s_ = 0.01 (p_corrected_ = 0.96)
sERT and number of correct answers	r_s_ = 0.23 (p_corrected_ = 1.00)	r_s_ = 0.12 (p_corrected_ = 1.00)	r_s_ = -0.06 (p_corrected_ = 1.00)	r_s_ = -0.06 (p_corrected_ = 1.0)	r_s_ = -0.04 (p_corrected_ = 0.86)
sERT and accuracy score	r_s_ = -0.10 (p_corrected_ = 1.00)	r_s_ = -0.19 (p_corrected_ = 1.00)	r_s_ = -0.08 (p_corrected_ = 1.00)	r_s_ = -0.27 (p_corrected_ = 1.00)	r_s_ = 0.08 (p_corrected_ = 0.73)
RTS and total number of answers	r_s_ = 0.34 (p_corrected_ = 0.50)	r_s_ = 0.22 (p_corrected_ = 1.00)	r_s_ = 0.08 (p_corrected_ = 1.00)	r_s_ = 0.02 (p_corrected_ = 0.91)	r_s_ = 0.18 (p_corrected_ = 1.00)
RTS and number of correct answers	r_s_ = 0.29 (p_corrected_ = 0.85)	r_s_ = 0.20 (p_corrected_ = 1.00)	r_s_ = 0.08 (p_corrected_ = 1.00)	r_s_ = -0.03 (p_corrected_ = 0.87)	r_s_ = 0.11 (p_corrected_ = 1.00)
RTS and accuracy score	r_s_ = -0.16 (p_corrected_ = 1.00)	r_s_ = -0.20 (p_corrected_ = 1.00)	r_s_ = -0.07 (p_corrected_ = 1.00)	r_s_ = -0.05 (p_corrected_ = 0.82)	r_s_ = 0.15 (p_corrected_ = 1.00)
Reps and total number of answers	r_s_ = 0.43 (p_corrected_ = 0.14)	r_s_ = 0.28 (p_corr_ = 0.37)	r_s_ = 0.51 (p_corrected_ = 0.05)	r_s_ = 0.23 (p_corrected_ = 0.28)	r_s_ = 0.34 (p_corrected_ = 0.30)
Reps and number of correct answers	r_s_ = 0.44* (p_corrected_ = 0.03)	r_s_ = 0.30 (p_corrected_ = 0.12)	r_s_ = 0.55 (p_corrected_ = 0.48)	r_s_ = 0.27 (p_corrected_ = 0.40)	r_s_ = 0.24 (p_corrected_ = 0.26)
Reps and accuracy score	r_s_ = -0.09 (p_corrected_ = 1.00)	r_s_ = 0.14 (p_corrected_ = 1.00)	r_s_ = 0.32 (p_corrected_ = 0.66)	r_s_ = 0.15 (p_corrected_ = 1.00)	r_s_ = 0.06 (p_corrected_ = 0.77)

## Discussion

The aim of this study was to quantify the amount of cognitive resources needed to perform resistance exercises in the form of low-load barbell back squats. To that end, the dual-task paradigm was applied. The observed behavioral performance costs during low-load barbell back squatting would allow to draw conclusions about the necessary higher cognitive resources [9–12]. Based on the observed decrease in total number of answer and number of correct answers in the DT condition (squatting while performing serial subtraction of 7’s), our results suggest that higher cognitive resources are required to perform low-load barbell back squats on a Smith machine. Furthermore, our results imply that the observed dual-task effect is rather quantitative than qualitative in nature because the accuracy of the answers remains unaltered. Our findings are in line with previously published studies reporting a significant decrease in cognitive performance (e.g., number of solved items) in DT conditions involving dynamic motor actions (e.g., walking) [42–44]. Such a decrease in cognitive performance in DT conditions could be explained by the ‘limited resource hypothesis’ which postulates that the pool of available cognitive resources is restricted [45,46]. In DT situations, the motor task and the cognitive task compete for limited cognitive resources. When the resources do not suffice in a way that the demands of both tasks are fully satisfied, this could lead to a decrease in the performance of the motor and/or cognitive task [45]. In the light of the limited resources hypothesis, our results suggest that the execution of low-load barbell back squats (as motor task) withdraw a considerable amount of cognitive resources (significant lower cognitive performance in conjunction with high effect sizes) from processes required to solve the cognitive task (serial subtraction of 7’s).

Furthermore, the positive moderate correlation between the number of squat repetitions and the number of correct answers in the first set suggests that some synchronization between the motor task and the cognitive task has occurred. Speculatively, the synchronization between motor tasks and cognitive tasks could be a strategy to better cope with the cognitive demands imposed by the simultaneous solving of specific motor task (i.e., squatting) and cognitive task (i.e., serial subtraction of 7’s) [47]. However, this finding should be threated cautiously as it (i) was not persistent across the second to fifth set and (ii) could not been observed regarding other parameters of cognition (i.e., total number of answers and accuracy score). Moreover, the observed DT effect cannot merely be a result of a higher physical exertion in the DT condition but rather a consequence of the execution of the low-load barbell back squat itself. This assumption is, at least partly, supported by the absence of statistically significant correlations between RPE scores or mean HR and measures of cognitive performance. Moreover, the absence of significant associations between resistance training experience and cognitive performance suggests that the observed dual-task effect is relatively independent of an individual’s expertise level in our study performing low-load barbell back squats on a Smith machine. This finding is in accordance with results of a previous study which did not report an effect of expertise level on cognitive performance changes in the dual-task conditions [48]. However, it is also reported that experts outperform novices in challenging dual-task conditions [49]. Hence, in order to rule out whether this finding is generalizable, further research should directly compare groups with different levels of expertise in resistance training and with different levels of load.

The observation that the number of total responses was significantly higher in the fifth set as compared to the first set indicates that some learning might have occurred. The appearance of a learning effect in DT conditions (e.g., with regard to cognitive measures) is in line with previous findings [50,51] and could be attributed to the automatization of task execution which leads to the freeing of cognitive resources [50]. However, given that the differences between ST and DT remained significant even in the fifth set, the presence of a supposed emerged learning effect would not argue against the assumption that higher cognitive resources are needed to perform low-load barbell back squats on a Smith machine. To further strengthen the assumption that higher cognitive resources are required to perform resistance exercises, more research is needed that investigates whether (i) dual-task effects emerge with other cognitive tasks that target other cognitive domains (e.g., working memory by n-back task), (ii) dual-task effects occur during the performance of other resistance exercises (e.g., seated rowing), or (iii) how dual-task effects are influenced by other exercise variables (e.g., load, rest phases, movement velocity). Additionally, as older adults require more generic resources to perform a motor task (e.g., postural tasks) [13], it seems promising to clarify in future research whether the observed dual-task costs in response to resistance exercises (i.e., barbell back squat) are more pronounced in the elderly.

## Limitations

While our results suggest that higher cognitive resources are necessary to perform barbell back squats, the findings need to be interpreted in light of some limitations. A drawback of this study is the abdication of kinematic analyses (e.g. by using motion capture systems) or muscle functional analyses (e.g., by using electromyography) of the barbell back squats. Such kinematic analyses or electromyographic analyses could be helpful to assess the motor-related dual-task costs and their application is recommended in further studies.

## Conclusion

In conclusion, our results suggest that in our cohort the execution of low-load barbell back squats requires the recruitment of higher cognitive resources. However, since our results are neither transferable to other cognitive tasks nor other cohorts, further studies which utilize other cognitive tasks (e.g., n-back task), conduct other resistance exercises (e.g., seated rowing), investigate a potential dose-response relationship (e.g., different loads), and/or recruit further cohorts (e.g., older adults, experts in resistance training) are necessary to confirm and generalize our findings.
